# Carob Pulp Flour as a Sustainable and Functional Ingredient in the Bakery: Effects of Leavening Typologies on Dough and Bread Properties

**DOI:** 10.3390/life15101571

**Published:** 2025-10-08

**Authors:** Sebastiano Rosati, Ilenia Gaeta, Lucia Maiuro, Maria Carmela Trivisonno, Maria Cristina Messia, Elena Sorrentino

**Affiliations:** 1 Department of Agricultural, Environmental and Food Sciences, University of Molise, 86100 Campobasso, Italy; s.rosati1@studenti.unimol.it (S.R.); mariacarmela.trivisonno@unimol.it (M.C.T.); messia@unimol.it (M.C.M.); sorrentino@unimol.it (E.S.); 2 Department of Agricultural, Forest and Food Sciences, University of Turin, 10124 Turin, Italy

**Keywords:** *Ceratonia siliqua*, carob pulp flour, sourdough, bread, starter cultures

## Abstract

Carob pulp flour (*Ceratonia siliqua*) is gaining attention as a sustainable ingredient with nutritional and functional potential. This study evaluated the partial replacement of soft wheat flour with 10% carob pulp flour in breadmaking, focusing on the role of different leavening strategies: commercial baker’s yeast (LB), a selected starter culture, *Lactiplantibacillus plantarum* SL31 and *Saccharomyces cerevisiae* SY17 (LI), and a type I sourdough (LS). Dough rheology, microbial dynamics, bread quality, acceptability, and shelf-life were assessed. Results showed that the inclusion of carob pulp flour enhances the nutritional profile while maintaining satisfactory technological performance. The leavening strategy strongly influenced the final products: breads made with commercial yeast displayed high volume and softness but were less stable during storage; LS breads achieved greater microbial stability but were limited by excessive acidity and reduced sensory acceptance; breads obtained with the selected starter culture offered the most balanced outcome, combining moderate structure with enhanced flavor and consumer preference. Overall, the findings demonstrate the feasibility of incorporating carob pulp flour into bakery products and highlight the potential of tailored starter cultures as a promising compromise between technological performance, sensory quality, and shelf-life. Future work should optimize fermentation approaches to further enhance consumer appeal and support industrial application.

## 1. Introduction

The increasing demand for sustainable and functional plant-based foods has driven the exploration of both traditional and non-traditional ingredients rich in bioactive compounds with proven health benefits. Within this context, carob (*Ceratonia siliqua*) has gained increasing scientific attention [1].

**Figure 1:**
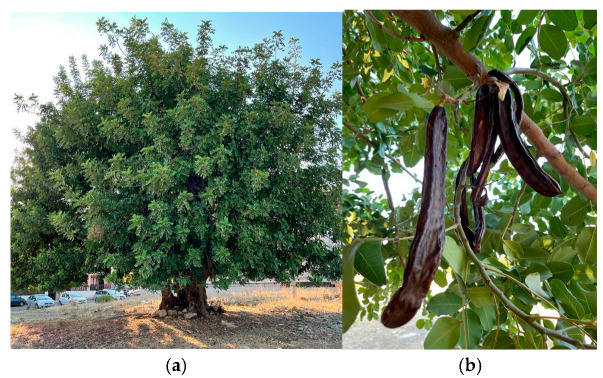
(**a**) Centuries-old carob tree located in the province of Taranto, Apulia region, Italy; (**b**) fruit at full ripeness.

Carob (*Ceratonia siliqua*) is native to the Mediterranean region and mainly found in Italy, Spain, Greece, Portugal, and Morocco [2]. The carob tree (Figure 1a) is an evergreen xerophytic species belonging to the Fabaceae family that produces large pods (Figure 1b). Its resilience to drought, poor soils, and harsh climatic conditions makes it an essential crop in the fight against desertification [1].

These pods were a common food in local communities not too long ago due to their nutritional value [3] but were later mostly used as animal fodder. In the context of the circular economy strategy, the food industry is currently showing increasing interest in the nutritional potential of the carob pulp.

Carob pulp is an interesting by-product of the industrial production of carob bean gum (E410) [4]. Dried and ground carob pulp is used to produce carob flour, a versatile food ingredient traditionally consumed in Mediterranean countries and now increasingly appreciated worldwide for its nutritional and functional properties [5,6,7,8]. Furthermore, carob is well-suited for cultivation in arid and marginal lands, requiring minimal irrigation and chemical input. Its cultivation contributes to reforestation and desertification control, supporting the transition to more sustainable agricultural systems. Valorization of carob by-products for food use also supports waste reduction, short and local supply chains, and the achievement of several United Nations Sustainable Development Goals (SDGs)—including Zero Hunger (Goal 2), Responsible Consumption and Production (Goal 12), and Climate Action (Goal 13).

Carob pulp flour is naturally gluten-free and therefore suitable for individuals with celiac disease. It is a good source of fibers, sugars, proteins, essential minerals, polyphenols (notably tannins), and other bioactive compounds, making it a promising candidate for the development of functional foods [1,9,10].

Over the past two decades, scientific interest has focused on the healthful components of carob, including dietary fiber, cyclitols, and polyphenols, which have been attributed to a wide range of beneficial effects, such as antioxidant, anti-inflammatory, and hypoglycemic properties [11]. Furthermore, carob extracts have shown strong antimicrobial activity against various bacteria and molds [12]. These characteristics suggest potential applications in promoting human health, including the management of gastrointestinal disorders and other chronic conditions [7,13,14]. Particular attention has been paid to the pulp for its potential to support the development of innovative food products in line with modern dietary needs and health trends [4,15].

Recently, the use of carob pulp flour, in combination with wheat flour, has grown in the Mediterranean bakery sector, driven by demand for more nutritious and sustainable ingredients, but technological challenges remain [1,16]. The incorporation of carob pulp flour into bread may affect dough fermentation, loaf volume, crumb texture, and sensory perception, depending on the fermentation process adopted. In particular, the choice between commercial yeast or sourdough strongly determines the extent of gas production, acidification, and metabolite release, which in turn modulate bread quality. While some studies have explored the impact of carob on breadmaking [1,3,15], few have systematically compared different fermentation approaches.

Given the growing interest in carob flour for its technological and nutritional potential, this study investigates its application in breadmaking through different fermentation strategies. Therefore, the aim of this research was to evaluate the effects of three leavening systems—(i) commercial baker’s yeast (LB), (ii) a selected starter culture composed of *Lactiplantibacillus plantarum* SL31 and *Saccharomyces cerevisiae* SY17 (LI), and (iii) Type I sourdough (LS)—on the characteristics of breads enriched with carob pulp flour.

## 2. Materials and Methods

### 2.1. Flour Samples

Carob flour, obtained from the pulp of *Ceratonia siliqua* pods, was provided by Industria Lavorazione Carrube ILCAR S.r.l. (Rosolini, Siracusa, Italy). Type “0” soft wheat flour was supplied by Molino Cofelice (Matrice, Campobasso, Italy). These flours were blended at 90:10 (wheat–carob) to obtain the CB10 blend.

#### 2.1.1. Microbiological Analysis of the Flours

For microbial counts, 10 g of each flour sample—carob flour, soft wheat flour, and the CB10 blend—were diluted in a sterile saline solution (0.9% NaCl), homogenized in a stomacher (Model BK-SHG04 BioBase—Osgood Common, Fremont, CA, USA) and serially diluted. The different microbial groups were enumerated using specific culture media and appropriate incubation temperatures. In detail, total mesophilic count (TMC) was performed on Plate Count Agar—PCA (Oxoid, Milan, Italy) incubated at 30 °C for 72 h; yeasts and molds on Dichloran Rose Bengal Agar (DRBA, Oxoid) at 28 °C for 24–48 h; lactic acid bacteria (LAB) on de Man, Rogosa, and Sharpe (MRS) agar (Oxoid) at 28 °C for 48 h in anaerobic conditions (in anaerobic conditions (GENbox anaer, bioMérieux, Marcy-l’Etoile, France)); Enterobacteriaceae on Violet Red Bile Glucose Agar (Oxoid) at 37 °C for 24 h; and *Bacillus cereus* on MYP (Biolife, Milan, Italy), added to selective supplements (Biolife), after 48 h of incubation at 30 °C. For the enumeration of *Bacillus* spores, the homogenized samples (first dilution) were subjected to heat treatment at 80 °C for 15 min, followed by rapid cooling in ice water, to inactivate vegetative cells, and then determined on PCA at 28 °C for 48 h. For the detection of *Salmonella*, 25 g of each sample were aseptically collected and diluted in 225 mL of pre-enrichment medium (Buffered Peptone Water) and incubated at 37 °C for 18 ± 2 h. Subsequently, an aliquot was transferred to the selective enrichment medium Rappaport Vassiliadis Broth (RVB, Oxoid) and incubated at 41.5 ± 1 °C for 24 ± 3 h. For confirmation, aliquots of the enrichment broth were streaked onto Brilliant Green Agar (Biolife) and incubated at 37 ± 1 °C for 24 ± 3 h.

#### 2.1.2. Chemical Analysis of Flours and Bread

Flour and bread moisture was determined according to ICC Method 110/1 [17]. Flour ash and protein content were determined according to ICC methods 104/1 and 105/2, respectively [17]. Flour fat content was assessed according to the AACC Method 30-20 [18]. Total dietary fiber was determined according to the AACC method 32-05.01 [18].

### 2.2. Starter Cultures

Three different starter cultures were used:

- **LS:** a Type I sourdough obtained through spontaneous fermentation. The LS starter was prepared using a back-slopping method reported by Eraslan et al. [19] with minor modifications. Briefly, 500 g of CB10 flour were mixed with water (according to the water absorption percentage determined by farinograph analysis), mixed and incubated at 28 °C for 15 days. The dough was refreshed every 5 days by adding the same flour blend and water. The final sourdough (LS) was either used in the leavening trials or stored at 4 °C for further use.
- **LI:** a selected starter culture consisting of *Lactiplantibacillus plantarum* SL31 and *Saccharomyces cerevisiae* SY17, both belonging to the Collection of the DiAAA (Department of Agricultural, Environmental and Food Sciences, University of Molise), and previously isolated from a spontaneously fermented carob pulp flour dough. These strains were selected for their technological properties, such as sugar fermentation activity, acidification and leavening ability, as reported by Messia et al. [20]. Prior to use, the microbial strains were revitalized in MRS broth (Oxoid) for SL31 and YPD for SY17. The overnight cultures were centrifuged (13,000 rpm for 15 min at 4 °C; Centrifuge 5415 R; Eppendorf, Hamburg, Germany), washed twice in 0.9% (*w*/*v*) NaCl solution, and used as inoculum.
- **LB:** commercial baker’s yeast (Lievital, © 2025 Lesaffre, Trecasali, Italy) used as control.

### 2.3. Dough and Bread Preparation

**Figure 2:**
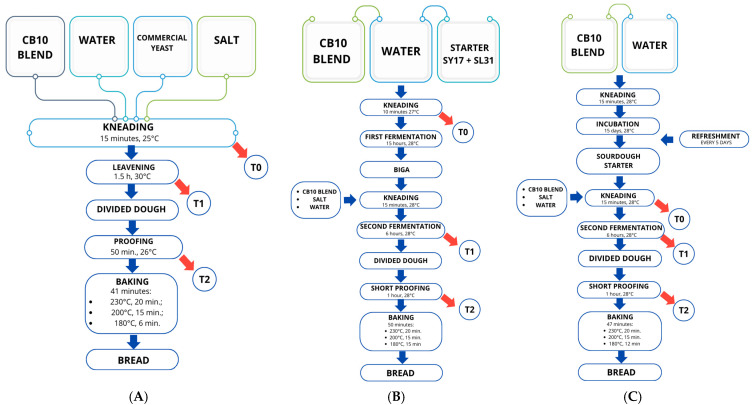
Breadmaking flowcharts. (**A**): direct method with commercial yeast (LB); (**B**): indirect method with selected sourdough starter (LI); (**C**): indirect method with sourdough starter (LS); and dough sampling scheme.

Three different batches were prepared using the CB10 blend, water, NaCl, and different leavening agents: LS, LI, and LB. A direct baking method was applied for the LB batch, whereas an indirect method was used for the LI and LS batches, employing a biga and a sourdough starter, respectively. The baking procedures adopted in the experimental trials are illustrated in Figure 2 and described in detail below.

*Direct dough method:*

- **LB:** CB10 blend (2 kg), salt (30 g), fresh baker’s yeast (50 g), and water (1130 mL, based on the absorption capacity determined by farinograph analysis) were mixed for 15 min in a planetary mixer (Conti, Bussolengo, Italy, mod. SP 20 2V). The leavening was carried out in a fermentation chamber at 30 °C for 90 min. The dough was then divided into loaves of approximately 300 g each, placed in aluminum trays, and subjected to a short proofing (50 min) at 26 °C. Baking was performed in a static electric oven (CIMAV, Villafranca, VR, Italy) for 41 min under the following conditions: 230 °C for the first 20 min, 200 °C for 15 min, and 180 °C for the final 6 min.

*Indirect method:*

- **LI:** CB10 blend (400 g) was mixed with water, adjusted according to farinograph absorption values, in which the selected strains SY17 and SL31 were suspended at approximate concentrations of ~4.5 and ~5.5 log CFU/mL, respectively. The dough was kneaded for 10 min in a planetary mixer and then subjected to a first fermentation in a fermentation chamber (15 h, 28 °C), thus obtaining a biga, a traditional Italian stiff pre-ferment. Afterwards, the flour blend (1600 g), water (adjusted to the absorption capacity determined by farinograph analysis), and salt (30 g) were added to the biga and mixed for 15 min in a planetary mixer. A second fermentation was then carried out in a fermentation chamber at 28 °C for 6 h. Each loaf (approximately 300 g) was shaped into aluminum trays and subjected to a final proofing step (1 h). Baking was performed in a static electric oven for a total of 50 min, under the following temperature profile: 230 °C for the first 20 min, 200 °C for 15 min, and 180 °C for the remaining 15 min.
- **LS:** A portion of sourdough (400 g), prepared as described in Section 2.2, was used as the starter. The procedure was the same as for the LI batch: CB10 blend (1.6 kg), water (according to the absorption capacity determined by farinograph analysis), and salt (30 g) were added to the sourdough and mixed for 15 min in a planetary mixer. Fermentation was carried out in a fermentation chamber at 28 °C for 6 h. Each loaf (approximately 300 g) was placed in aluminum trays and subjected to a final short proofing (1 h). Baking was performed in a static electric oven for 47 min under the following temperature profile: 230 °C for the first 20 min, 200 °C for 15 min, and 180 °C for the final 12 min.

After baking, the loaves were left to cool at room temperature for 2 h before further analysis. Some loaves from each batch were packaged in paper bags for bread storage.

### 2.4. Dough Analyses

During leavening, the pH was measured by directly inserting the probe of a pH meter (Hanna Instruments, Padova, Italy) into the doughs. The pH measurement was carried out for each batch at three different time points as reported in Figure 2: immediately after preparation (T0), after the first leavening for LS and LB and after the second leavening for LI (T1), and just before baking (T2).

At the same time points, the doughs were subjected to microbiological analysis to assess yeast and LAB counts using the viable plate count method previously described (Section 2.1.1), with the only difference that YPD was used as the substrate for the yeast counting (Sigma Aldrich, St. Louis, MO, USA).

Farinograph and Alveograph analyses were carried out according to AACC International Methods (54–21.01 and 54–30.02, respectively) [18]. The Chopin alveograph (Belotti Strumenti srl, Peschiera Borromeo (MI), Italy) was used to determine the viscoelastic properties of the dough subjected to mechanical processing for evaluating the dough tenacity (*p*-value), swelling index (G value), dough extensibility (L value), flour strength (W value) and the ratio between tenacity and extensibility (P/L value). The Brabender farinograph (Brabender, Duisburg, Germany) was used to determine water absorption, development time, stability, and degree of softening.

### 2.5. Breads Analyses

#### 2.5.1. Color

The crust and crumb color of the experimental breads were evaluated using a colorimeter (CR-300 Minolta, Osaka, Japan). The color of raw materials was expressed in the CIE L*a*b* color system [21] through coordinates: L*—lightness of color, a*—redness (+) or greenness (−), b*—yellowness (+) or blueness (−). The total color variation (ΔE) was determined by comparing each experimental bread sample (LI and LS) to a reference yeast sample (LB). In the specific formula applied as follows,

$$\Delta E=\sqrt{(L_{1}^{*}-L_{2}^{*}{)}^{2}+(a_{1}^{*}-a_{2}^{*}{)}^{2}+(b_{1}^{*}-b_{2}^{*}{)}^{2}}$$

the subscripts 1 and 2 refer to the reference yeast bread (LB) and the other experimental sample being evaluated (either LI or LS), respectively.

#### 2.5.2. Consumer Preference Evaluation

A consumer acceptability test was carried out 24 h after baking with 15 untrained participants (9 female and 6 male, aged 20–60 years), all bread consumers affiliated with the Department of Agricultural, Environmental and Food Sciences (University of Molise), including professors, technical staff, PhD students, and students. Bread samples were coded with 3-digit random numbers and presented in randomized order [22]. A ten-point hedonic scale (1 = extremely dislike, 5 = neither like nor dislike, 10 = extremely like) was used to evaluate consumer perception of the main product attributes: crust color and appearance, crumb firmness, aroma, flavor, sweetness/acidity (sweetness = high score, acidity = low score), and overall acceptability. Participants were provided with fresh water for palate cleansing between samples. This approach was intended as a consumer-oriented evaluation of bread acceptability rather than a formal sensory descriptive analysis.

### 2.6. Bread Characterization During Storage

Samples from each batch were packaged in paper bags and stored at room temperature for 7 days. Analyses were performed 16 h after baking (t1), on day 3 (t3), and on day 7 (t7) of storage.

Water activity of breads was measured using the AquaLab 4TE instrument (Addium Inc., Pullman, WA, USA). From each test loaf, four 1-cm-thick slices (two from the ends and two from the center) were collected, cut into smaller pieces, and analyzed.

Bread weight (g) was determined on each day of analysis by weighing the entire loaf. The specific volume of bread (measured in mL/g) was measured according to the rapeseed displacement method [23]. This value was calculated as the ratio of loaf volume and corresponding loaf weight.

Texture analysis was carried out according to AACC 74-09 [24] using a texture analyzer TA-XT2i (Stable MicroSystem, Godalming, UK), equipped with a 5 kg loading cell and a P/36R probe, under the following settings: pre-speed 1.0 mm/s; test speed 1.0 mm/s; post-speed 1.0 mm/s; distance 30.0%; rupture test distance 4.0%; force 10.0 g; temperature 25 °C. Samples were constituted by slices of bread with a thickness of 25 mm, and the measurements were carried out on the central part of the slice.

At t7, a central slice of approximately 10 g was aseptically taken from the breads. Samples were diluted 1:10 (*w*/*v*) with sterile physiological solution (0.9% NaCl) and homogenized. Spores of *Bacillus* spp. and molds were determined as previously described.

### 2.7. Statistical Analysis

All reported results are expressed as the mean ± standard deviation (SD), based on three independent replicates. Statistical analysis was conducted using SPSS software (version 22.0; IBM SPSS Statistics, Armonk, NY, USA). Depending on the dataset, either a one-way ANOVA or a repeated-measures ANOVA was performed, followed in both cases by Tukey’s post hoc test. A *p*-value < 0.05 was considered statistically significant for all analyses.

## 3. Results and Discussion

The carob pulp flour used in this study, as stated by the manufacturer, is obtained from milling the whole carob pod. Appreciated for its pleasant flavor and high carbohydrate content, as well as its notable levels of dietary fiber, minerals, and polyphenolic compounds, carob pulp flour represents a nutritionally rich ingredient of considerable interest [11].

Partial replacement of wheat flour with carob flour alters the rheological properties of doughs. Turfani et al. [25] produced doughs that were more tenacious and stable, with higher water absorption requirements. This behavior contrasts with that observed when incorporating other legume flours (e.g., lentil flour), which generally reduce dough tenacity, extensibility, and strength. Furthermore, dough development time tends to be longer when carob flour is included, a trend similarly observed with other legume flours, including lentil and pea [26]. Overall, wheat–carob blends influence the technological performance of bread, with potential benefits or disadvantages depending on the level of carob flour concentration [25]. On the other hand, carob pulp flour seems to be a suitable ingredient for enriching wheat bread (especially for nutritional enhancement), despite drawbacks regarding starch gelatinization or water distribution between the starchy and protein fractions, including gluten [3].

For these reasons, in the present study, the replacement level of wheat flour with carob flour was set at 10% to minimize the issues commonly associated with adding non-conventional gluten-free ingredients to bread formulations. The quantity and quality of gluten-forming proteins are key factors determining the technological performance of wheat flour, especially in terms of dough extensibility, elasticity, and overall strength. Gluten is essential for retaining the gas produced during fermentation and for contributing to the development of dough structure and bread texture. A reduced gluten content or an impaired gluten network typically leads to breads with lower loaf volume, firmer crumb, and diminished palatability, which may negatively impact visual and sensory consumer acceptance.

In this context, evaluating the effects of adding 10% carob pulp flour on both dough rheology and bread quality becomes crucial to balancing the nutritional benefits with technological performance. The following results present the impact of different leavening strategies on these parameters, allowing a direct comparison with the technological trends reported in the literature.

### 3.1. Chemical and Rheological Properties of Flours

Table 1 reports the chemical composition of wheat flour, carob pulp flour, and the CB10 blend (90% wheat flour + 10% carob flour) used in this study.

For its chemical features, the wheat flour met the requirements established by Italian regulatory standards [27].

The chemical composition of the carob pulp flour aligned well with values reported in the literature [28,29], confirming its nutritional and functional potential. Its moisture content (4.1%) fell within the lower range documented for dried carob pulp flour, reflecting its naturally low-moisture character and good storage stability. Also, the protein content (4.9%) was comparable to literature values (6.48% in Khelouf [28]; 5.90% in Petkova [29]).

In particular, the ash content (2.96%) confirmed carob flour as a good source of minerals. Literature reports indicate that carob pulp is particularly rich in calcium, potassium, and magnesium, along with appreciable amounts of phosphorus and trace elements such as iron, copper, manganese, and zinc [28,30]. These minerals are relevant not only from a nutritional standpoint but also for their potential influence on yeast and LAB metabolism, dough rheology, and crust coloration during baking. For example, calcium and magnesium can influence enzyme activity and gluten interactions, while potassium is known to play a role in yeast fermentation performance.

The fat content (5.6%) was slightly higher than some literature values and significantly higher (*p* < 0.05) than in wheat flour (1.6%), possibly due to residual seed particles or processing methods that retain more lipids. The dietary fiber content (31%) was significantly higher, fully supporting its classification as a fiber-rich functional ingredient and in line with the 33–40% range reported in previous studies, though variations in analytical methods may explain discrepancies. Consequently, the carbohydrate content (51.44%, calculated by difference) was lower than that of wheat flour but still consistent with its characterization as a carbohydrate-rich ingredient containing a substantial proportion of natural sugars.

The CB10 blend, composed of 90% wheat flour and 10% carob pulp flour, exhibited intermediate properties: moisture and protein levels remained similar to those of wheat flour, although protein was slightly reduced (11%, *p* < 0.05), potentially affecting gluten development. The blend maintained a low-fat content (2%) but showed a marked and statistically significant increase in dietary fiber (+93% compared to wheat flour, *p* < 0.05) and ash (+53%, *p* < 0.05), highlighting the nutritional improvement achievable through the incorporation of carob. In particular, the mineral levels in the CB10 blend may improve micronutrient density and may contribute positively to yeast performance and dough structure. At the same time, the higher fiber content can influence water absorption, dough handling, and gluten network formation. The higher mineral content (ash) and the presence of bioactive compounds in carob pulp flour, particularly phenolics with antimicrobial activity, may also have influenced microbial growth during fermentation, as discussed in Section 3.2. Moreover, the naturally low moisture content of carob pulp flour could contribute to improved raw material storage stability, an aspect relevant to industrial applications.

The rheological properties of dough prepared with the CB10 blend were evaluated by farinograph and alveograph analyses (Table 2). Considering the W values (flour strength) and the P/L ratio (tenacity/extensibility balance), the CB10 blend displayed rheological performance typical of medium-strength flours suitable for breadmaking. Although carob pulp flour alone lacks the viscoelastic gluten network required for mechanical handling and long fermentation [11], its low inclusion level (10%) preserved most of the functional characteristics of wheat flour, which is traditionally preferred for indirect breadmaking.

The farinograph indicated that 56.5% water was needed to reach optimal dough consistency, suggesting that this substitution level does not significantly alter hydration requirements. The slightly higher ash content of CB10, reflecting its increased mineral content, may have contributed to its rheological performance. Calcium and magnesium ions are known to interact with gluten proteins, reinforcing the network, while potassium and other salts can modulate yeast activity and fermentation kinetics. The combined effect of these minerals, along with the dilution of gluten due to partial wheat flour replacement, resulted in rheological values that remain within optimal limits for breadmaking [31].

Overall, these findings highlight the dual role of carob pulp flour as a nutritionally enriching and technologically viable ingredient, enhancing mineral and dietary fiber content while maintaining satisfactory rheological properties when used at a 10% substitution level.

### 3.2. Microbiological Aspect of Flours, Starters and Doughs

Both wheat and carob pulp flours, as well as the CB10 blend, showed excellent microbiological quality: TMC < 100 CFU/g; yeasts, molds, LAB, and Enterobacteriaceae < 10 CFU/g. No *Salmonella* spp. or *Bacillus cereus* was identified in any of the samples. Notably, spores of *Bacillus* spp., among the main spoilage agents in baked products responsible for the “ropy bread” defect, were also undetectable.

The excellent microbiological quality of wheat flour was expected due to the high-grade supplier. For carob pulp flour and CB10 blend, the low microbial counts may also be attributed, at least in part, to the antimicrobial activity of phenolic compounds naturally present in carob pulp flour [28]. For example, Zahorec et al. [12] found significant amounts of gallic acid, quercetin, and other polyphenols in carob, which have been known to exert antimicrobial effects against a variety of microorganisms by interfering with cell wall integrity and enzyme activity [12,32].

These results for carob flour are consistent with findings reported by Mom et al. [33]. Regarding starters, strains SL31 and SY17, used as starters in the LI batch, were selected for their technological properties and belong to species commonly employed as selected starters in bread production. Both *Lactiplantibacillus plantarum* and *Saccharomyces cerevisiae* are widely found in sourdoughs [34,35].

*Lpb. plantarum* is often the dominant homofermentative LAB species, while *S. cerevisiae* is typically the main yeast isolated during the production of Type I sourdoughs [34,36]. Similarly, Sanmartín et al. [2], in a study on the effects of Type IV sourdough fermentation on carob flour, microbial dynamics, and technological properties, reported that *Lpb. plantarum* was the dominant LAB in carob and carob–wheat blend sourdoughs, and *S. cerevisiae* was the most abundant yeast in carob–wheat sourdoughs.

**Figure 3:**
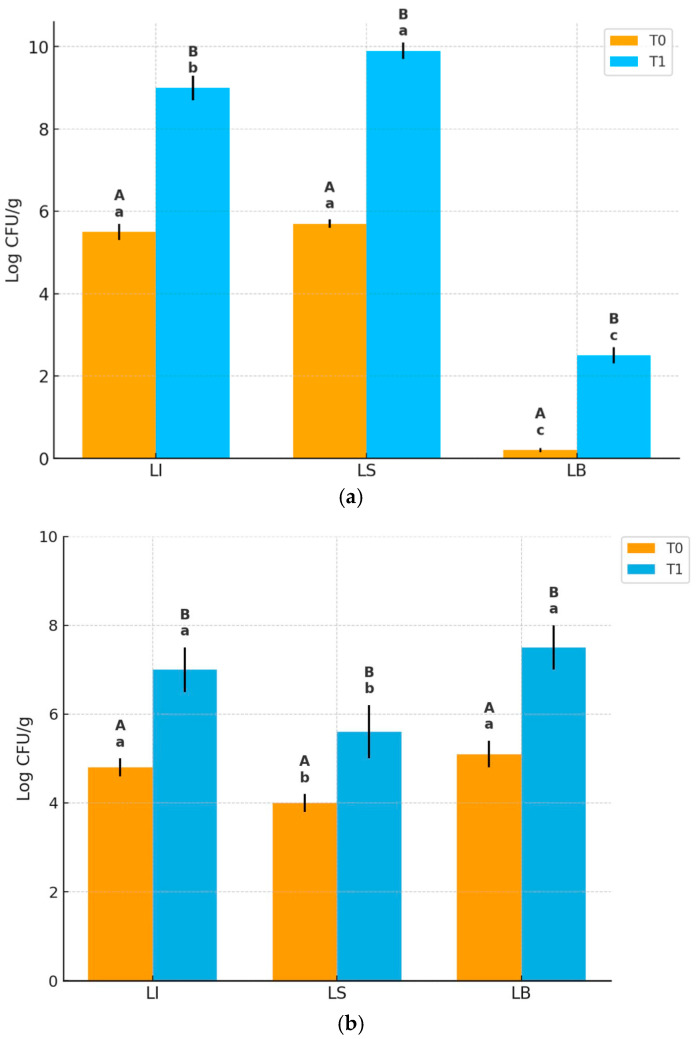
Microbial counts (log CFU/g) of (**a**) LAB and (**b**) yeasts in the three dough formulations (LI, LS, LB) at T0 and after the first fermentation (T1). Results are expressed as mean ± SD (n = 3). Different lowercase letters indicate significant differences among dough types at the same time point (*p* < 0.05). Different uppercase letters indicate significant differences between T0 and T1 within the same bread type (*p* < 0.05).

As shown in Figure 3, at T0, LAB counts were similar in LI and LS (~5.6–5.9 log CFU/g, while LAB in LB were undetectable. After fermentation (T1), LAB counts significantly increased in LI (9 log CFU/g) and LS (10 log CFU/g), with LS reaching significantly higher levels than LI; LAB in LB also increased but remained low (~2 log CFU/g).

Yeast counts at T0 were significantly higher in LI and LB (~4.7–5.0 log CFU/g) compared to LS (~4 log CFU/g). After fermentation (T1), yeast counts increased in all samples reaching ~7 log CFU/g in LI and ~7.5 log CFU/g in LB, and a lower level (~5.5 log CFU/g) in LS. Therefore, although yeast growth occurred in all batches, LB showed the highest yeast concentration, followed by LI, with LS significantly lower. No significant changes in yeast and LAB counts occurred between T1 and T2, but the further decrease in pH values at T2 (Table 3) suggests ongoing microbial metabolic activity.

Focusing on the individual batches, in the LI dough, LAB and yeast concentrations at T0 were consistent with the inoculation levels of strains SY17 and SL31, as previously described (Section 2.3). Then, as expected, microbial counts increased, reaching approximately 9 log CFU/g for LAB and 7 log CFU/g for yeasts at T1.

Regarding the LS dough, it can be hypothesized that the endogenous microbiota of the CB10 blend already included LAB and yeasts, although they were not detectable during the microbiological analyses of the flours (Section 3.2). These microorganisms likely found favorable conditions for growth during the fermentation steps, resulting in particularly high LAB concentrations. The microbiological data obtained for LS dough are consistent with results reported by Karlıdağ et al. [37], who investigated the influence of sourdough on the rheological properties of doughs prepared with various concentrations of carob flour. In the LB dough, at T1, LAB were also identified, albeit at a relatively low concentration (~3 log CFU/g), despite the exclusive use of commercial yeast. This finding aligns with those of Reale et al. [38], who reported the presence of LAB in several commercial yeast samples in a study assessing their microbiological quality.

### 3.3. Evolution of the pH of Doughs and Breads

Table 3 summarizes the pH changes in LB, LI, and LS doughs during fermentation and in their corresponding baked breads. At T0, LS and LI showed comparable initial pH values, significantly lower than LB. During leavening, a progressive decrease in pH was observed in all batches, but with different intensities depending on the fermentation method.

At T1, after the first fermentation step, LB still retained the highest pH (5.21), while LI and LS showed significantly lower values (4.65 and 4.55, respectively). Acidification became more marked at T2, where LB exhibited only a modest reduction (5.06), remaining significantly higher than LI (4.05) and LS (3.75). Among the indirectly fermented doughs, LS was the most acidified, with a final pH drop of 1.37 units from T0 to T2 (*p* < 0.05), compared with a decrease of 1.06 units in LI and only 0.28 units in LB.

In the baked breads, the same pattern persisted: LB retained the highest pH, LI showed an intermediate value, and LS reached the lowest value, with all differences being statistically significant (*p* < 0.05). This indicates that the acidification achieved during fermentation was effectively preserved in the final product.

The stronger acidification of LS is consistent with the higher LAB population identified in this batch (Figure 3), confirming the role of lactic acid bacteria as the main producers of organic acids in sourdough systems. This pronounced pH decrease translated into higher perceived acidity during sensory evaluation. In contrast, in LI bread, the metabolic activity of the yeast SY17 appeared to partially buffer the acidification induced by *Lpb. plantarum* SL31, resulting in an intermediate final pH.

The lower pH values in LS and LI compared with LB are also relevant for bread quality, since higher acidity is known to limit Maillard browning precursors and to contribute to improved microbial stability during storage, particularly by inhibiting the development of rope spoilage bacilli [39]. Comparable acidification dynamics in carob-enriched sourdoughs have been reported by Sanmartín et al. [2] and Zahorec et al. [12]. although the extent of the pH decrease is strongly dependent on starter composition and fermentation time.

### 3.4. Breads Analyses

**Figure 4:**
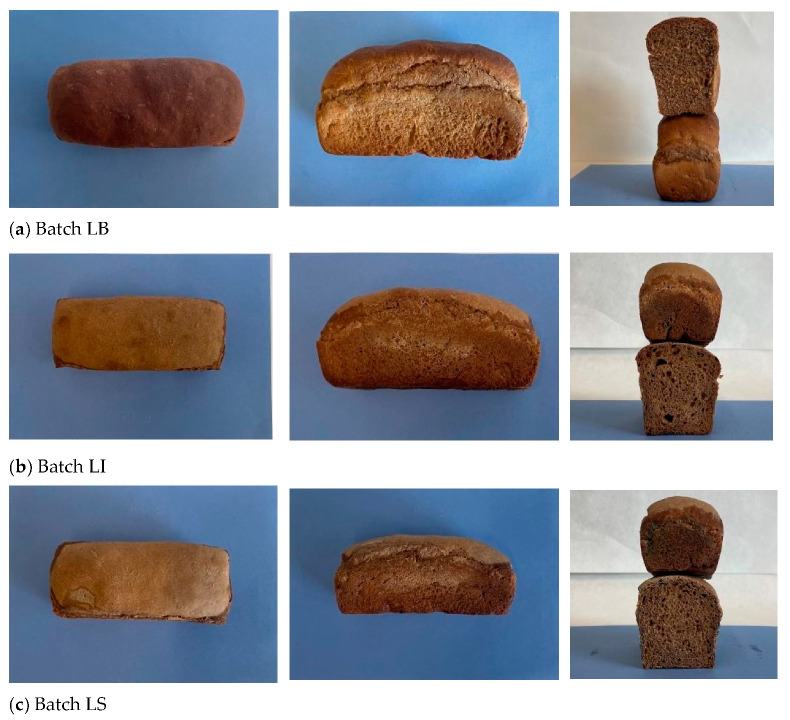
Images of the experimental bread batches: (**a**) LB; (**b**) LI; (**c**) LS.

The different experimental breads (LB, LI, and LS), produced using the CB10 blend, are presented in Figure 4.

Figure 4 highlights the differences in crust color, loaf volume, and crumb structure among the breads at the end of baking.

Table 4 reports the colorimetric indices (L*, a*, b*, chroma C*, and hue angle h°) and total color variation (ΔE) of the crust and crumb of the experimental breads. Overall, all breads exhibited relatively low lightness (L*) values, indicative of limited perceived brightness, which is consistent with expectations given that the CB10 flour blend used in the formulations possesses an intrinsically dark color due to the inclusion of carob flour [40]. Zahorec et al. [12] likewise observed that increasing the addition of carob pulp extract led to a progressive decrease in crust L* and an increase in a*, attributable to the intrinsic dark-brown pigments of carob, particularly tannins.

The crust colorimetric profile (Table 4) showed statistically significant differences (*p* < 0.05) among breads. LB bread exhibited the lowest lightness (L* = 36.90 ± 1.84), together with the highest a* (13.93 ± 0.49) and lowest b* (18.17 ± 1.57), corresponding to a darker reddish-brown crust. In contrast, LS (55.88 ± 2.18) and LI (48.26 ± 1.77) displayed significantly higher L* values, indicating a paler crust. The lighter color of sourdough bread (LS) is consistent with the reduced Maillard browning typically associated with sourdough fermentation, where the combined effect of lower pH and microbial metabolism reduces the availability of unprotonated amino groups and free sugars, both of which are critical precursors of Maillard reaction products [39]. Moreover, the L* value observed in LS is in line with that reported by Novotni et al. [40] for sourdough breads supplemented with carob flour.

The differences in crumb color were less pronounced and, as also highlighted by the ΔE value, similar between LI and LS compared to LB. LB showed the highest L* (49.37 ± 2.09), while LI (44.10 ± 1.15) and LS (44.00 ± 2.46) were significantly darker (*p* < 0.05). This reduction in lightness for indirect-method breads may be related to longer fermentation and acidification, which enhance pigment solubilization and possibly oxidative reactions of phenolic compounds, yielding a more uniform but darker crumb. Zahorec et al. [12] also reported similar trends, where carob extract addition led to reduced crumb lightness and increased a* values, again attributed to tannins and other intrinsic pigments of carob pulp. Yellowness (b*) values followed the order LB > LI ≈ LS, consistent with pigment dilution effects from the carob flour. Hue angle (h° ≈ 68°) remained stable across samples, indicating a predominance of yellowish tones irrespective of fermentation type.

Overall, at a 10% inclusion level, carob flour affects crust color more strongly than crumb, while the type of starter influences the extent of browning and yellow hue development. The bread produced with commercial yeast (LB) exhibited the greatest volume (Table 5) and a crumb characterized by a fine and uniform alveolar structure, in contrast to the coarser and less homogeneous crumb observed in LI and LS breads.

### 3.5. Consumer Preference Evaluation of Experimental Breads

For the development of new products with improved nutritional and sustainability profiles, often differing from traditional ones, it is crucial that changes in the parameters defining sensory quality remain within acceptable limits. This ensures broad consumer acceptance and promotes the integration of functional components into the diet [15]. For this reason, the experimental breads were evaluated to ensure their consumer acceptability.

**Figure 5:**
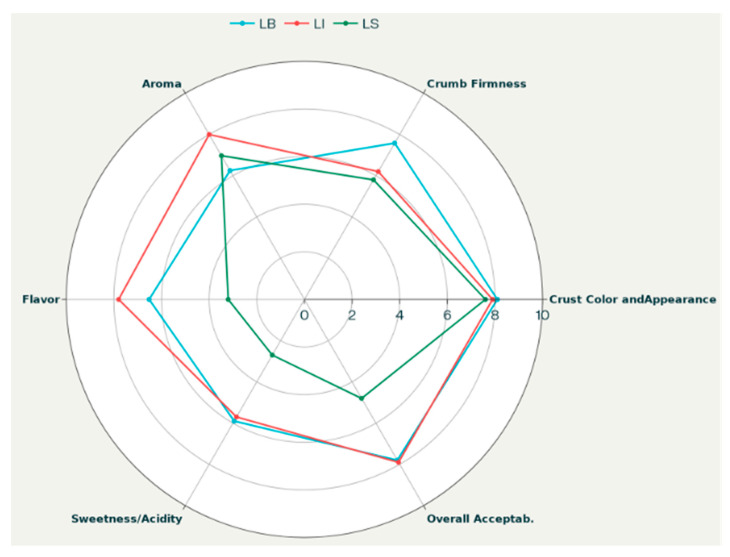
Consumer preference evaluation of experimental breads.

The radar chart in Figure 5 highlights the different degrees of consumer preference for the three bread batches (LB, LI, and LS), which are consistent with their physicochemical, colorimetric, physical, and textural characteristics, the last two of which are discussed below.

LB bread, exhibiting the highest loaf volume and moisture content, also scored highest for overall acceptability, crumb firmness, and crust color. These positive sensory results align with its higher pH (>5), contributing to a milder acidity favored in taste, and its softer crumb texture, as indicated by lower hardness and gumminess in the TPA data. The darker and reddish-brown crust coloration (lower L* and higher a*) further enhances the appealing crust appearance, complementing the overall sensory evaluation. However, LB bread received lower scores for aroma and taste, likely due to the exclusive use of commercial yeast and the direct baking method, which involves a much shorter leavening time and limits yeast metabolic activity. In contrast, LB achieved the highest scores for crumb development, reflecting its softness, uniformity, and perceived sweetness (Table 4 and Table 5). This superior crumb development can be attributed to the optimal yeast performance in LB, where the absence of LAB competition allowed for more effective yeast fermentation and CO_2_ production, positively impacting loaf volume and crumb structure.

Conversely, LS bread exhibited a markedly lower pH (3.68), due to pronounced LAB activity during sourdough fermentation. This strong acidification was reflected in low sweetness scores (high acidity) and low overall acceptability, indicating that excessive acidity negatively affected consumer evaluation. LS also exhibited a firmer crumb and reduced moisture retention compared to LB, as indicated by higher hardness, gumminess, and chewiness values from TPA analysis. The more compact crumb structure may be partially attributed to this elevated acidity, as further discussed in the next section. Nevertheless, LS bread achieved good scores for crust color and aroma, underlining the positive contribution of sourdough metabolites to flavor complexity. LI bread, fermented with the selected starter culture, exhibited an intermediate profile between LB and LS for several parameters. These characteristics correspond to a moderate pH (approximately 4.12), moderate moisture content and loaf volume, and intermediate TPA texture values (Table 5 and Table 6). For other attributes, such as aroma and flavor, LI achieves the highest scores, likely due to the synergistic metabolic activities of the SL31 and SY17 starter strains. This balance between sensory and physicochemical properties likely contributed to LI receiving the highest overall sensory acceptability, reflecting an optimal compromise among acidity, volume, and textural softness. Statistical analysis using one-way ANOVA followed by Tukey’s post hoc test (*p* < 0.05) confirmed that the observed differences between the bread types were significant for pH, moisture content, loaf volume, specific volume, and key TPA parameters.

### 3.6. Changes in Physical, Textural and Microbiological Parameters During Storage

Table 5 reports the evolution of crumb moisture content, water activity (aw), loaf weight, loaf volume, and specific volume of the bread batches over 7 days of storage at room temperature. Results are expressed as mean ± SD and include absolute (Δ) and percentage (%Δ) changes between day 1 (t1) and day 7 (t7). Lowercase superscripts indicate significant differences (*p* < 0.05) over time within the same batch; uppercase superscripts indicate differences between batches at the same time (ANOVA, Tukey’s HSD). Analysis of moisture content, water activity, weight, volume, and specific volume over the storage period (t1–t7) revealed significant changes within each bread batch and relevant differences between formulations.

At t1, moisture contents were comparable among batches, with no statistical differences between LB and LS and slightly lower values for LI. Over storage, all batches showed significant moisture decreases (LB: −26.4%, LI: −28.4%, LS: −28.8%), in line with water migration and evaporation during staling [41]. Weight loss closely mirrored moisture changes, being highest in LB and lowest in LS (statistically significant at t7).

Water activity declined slightly in all samples over 7 days, from 0.91–0.94 at t1 to 0.85–0.89 at t7. Significant within-batch reductions were observed for LB and LS (−6.4% and −6.6%, respectively), while LI remained stable and did not differ significantly from its initial value. At t7, LS showed a significantly lower aw than LB and LI; however, the values for all batches remained well above the mold growth inhibition threshold (0.80) [42]. Since molds are the main spoilage agents of baked products, these results confirm that water availability cannot be considered a limiting factor for the risk of microbial spoilage in bread. Although water activity values remained favorable for microbial growth, mold levels were less than 100 CFU/g, and *Bacillus* spp. were undetectable in all batches. These findings suggest that factors other than water availability, such as formulation and the increased presence of phenolic compounds with antimicrobial activity, previously discussed in connection with the addition of carob flour, played a key role in limiting microbial spoilage [12,28].

The inclusion of phenolic-rich flours such as carob can lead to a denser crumb and lower loaf volume due to interactions between phenolic compounds and gluten proteins that compromise the dough’s gas retention capacity [43,44]. Indirect fermentation methods, including sourdough or selected starter cultures, tend to exacerbate this effect, yielding more compact loaves than direct yeast fermentation. At t1, LB breads, produced with commercial yeast, exhibited significantly higher loaf and specific volumes compared to LI and LS (*p* < 0.05), reflecting greater gas production and subsequent expansion. This finding is consistent with [45], who reported that yeast-only leavening promotes higher initial gas retention and loaf volume. In contrast, loaves from LI and LS were more compact, attributable not only to gluten dilution and phenolic–protein interactions from carob flour but also because of the elevated crumb acidity generated during prolonged fermentation. This pronounced acidification can enhance protease activity, the gluten network and consequently reduce CO_2_ retention capacity [46,47]. These mechanisms could explain the lower volumes of LS and LI compared to LB.

During storage, all breads showed a progressive decline in weight, paralleling moisture loss, but the extent of change differed among batches. LB breads underwent the greatest reduction (−11.8% from t1 to t7), significantly higher than LI (−10.8%) and LS (−8.8%). The lower weight loss in LS, despite moisture losses comparable to LI, suggests a greater retention of bound water, likely due to the water-binding capacity of carob fibers and the structuring effect of sourdough metabolites. Volume evolution revealed the most striking differences. LB loaves decreased sharply from 667 mL at t1 to 507 mL at t7, corresponding to a significant shrinkage of −24.0% (*p* < 0.05). By contrast, LI and LS showed more moderate volume losses (−10.1% and −9.9%, respectively). Consequently, while LB had the largest initial volume, it exhibited the greatest structural collapse over time.

Specific volume trends confirmed this pattern. LB showed a significant decrease from 2.21 to 1.90 mL/g (−14.0%), whereas both LI and LS maintained stable specific volumes throughout storage (Δ ≤ ±1%, not significant). These findings indicate that indirect fermentation and sourdough contributed to preserving crumb porosity and preventing excessive collapse, even in the presence of carob flour.

The combined analysis of weight, volume, and specific volume highlights a trade-off between initial loaf expansion and structural stability during storage. Direct yeast fermentation (LB) produced loaves with superior initial volume but markedly lower stability, while sourdough (LS) and selected starter breads (LI) achieved more modest initial expansion but preserved their loaf shape and crumb structure significantly better during storage. This resilience has been attributed to the combined effect of acidification, microbial and endogenous enzymatic activities and the production of exopolysaccharides, which together mitigate staling [48].

The texture parameters evaluated over 7 days enable monitoring of the staling phenomenon in the experimental breads (Table 6). At t1, LB breads showed the lowest hardness (≈76 g) compared to LI and LS (≈165–173 g), reflecting a softer crumb and consistent with their higher loaf volume. During storage, hardness increased significantly in all samples, particularly in LI (up to ~424 g) and LS (~376 g), whereas LB remained markedly softer (~145 g at t7). Springiness and cohesiveness declined over time, with the sharpest decrease observed in LB, while LI and LS retained higher springiness but displayed less cohesive crumbs. Gumminess and chewiness were higher in LI and LS from the beginning and further increased during storage, while LB maintained consistently lower values. Overall, LB breads preserved a softer and less gummy crumb structure, whereas LI and LS developed denser, chewier textures over storage.

The evolution of textural properties (TPA, Table 6) can be interpreted in close connection with the changes in moisture, water activity, loaf weight, and volume (Table 5). At t1, moisture content was comparable among batches; however, LB exhibited significantly lower firmness (75.9 g) compared to LI and LS (>160 g). This softness is consistent with the higher loaf volume and specific volume observed in LB, reflecting greater gas expansion associated with direct yeast fermentation [45]. In contrast, LI and LS loaves, which had lower initial volumes, showed significantly firmer crumbs (*p* < 0.05). This finding is consistent with Song et al. [45], who reported that fermentation involving lactic acid bacteria results in reduced CO_2_ production compared to direct yeast fermentation, due to extensive protein hydrolysis that disrupts the gluten–starch matrix and compromises gas retention, thereby limiting dough expansion.

Over storage, all breads underwent a moisture loss (−26 to −29%) and corresponding weight reduction (−9 to −12%), but the structural consequences differed depending on the fermentation process [41]. LB underwent the largest decline in loaf volume (−24.0%) and a significant reduction in specific volume (−14.0%), followed by a sharp increase in hardness (+92%). This pattern indicates that the more expanded crumb structure of LB was less resistant to water loss and mechanical collapse, leading to faster firming and chewiness changes despite its initially softer texture. By contrast, LI and LS breads retained significantly more stable volumes (−10.1% and −9.9%) and specific volumes (not significantly different from t1), but their crumb hardness increased much more steeply, especially in LI. This suggests that although the denser structure of indirect method breads resisted shrinkage, water redistribution and interactions with fiber and proteins accelerated crumb firming, resulting in higher hardness and chewiness values than LB at the end of storage. Springiness and cohesiveness trends support this interpretation. LB lost elasticity and structural integrity (springiness −20%, cohesiveness −38% at t3), consistent with its collapse in volume. LI and LS maintained higher springiness but showed reduced cohesiveness. The relative stability of LS and LI compared with LB may be attributed to LAB metabolisms, including exopolysaccharide production and enhanced water binding by carob fiber fractions, which contribute to maintaining crumb elasticity and limiting excessive collapse.

Taken together, these results highlight a trade-off: breads produced with direct yeast fermentation exhibit greater initial softness and volume but lower stability during storage, whereas indirect processes (sourdough and selected starter) enhance structural resilience at the cost of higher firmness. Therefore, since this study did not identify the optimal starter for bread made by replacing 10% of the soft wheat flour with carob pulp flour, future optimization may involve combining the gas-producing efficiency of commercial yeast with the acidification and metabolite-driven structural reinforcement of selected starters (e.g., *Lpb. plantarum*), to achieve a balance between loaf expansion, softness, and storage stability.

## 4. Conclusions

Partially replacing wheat flour with 10% carob pulp flour effectively enhances the nutritional profile of bread without compromising its key technological properties.

The leavening strategy emerged as the main determinant of bread quality: commercial yeast ensures high loaf volume and a soft crumb but reduces storage stability, whereas sourdough improves microbial resilience at the expense of sensory acceptability. The selected starter culture (*Lpb. plantarum* SL31 + *S. cerevisiae* SY17) provides the best compromise, balancing flavor complexity, shelf-life, and texture. These findings suggest that using carob pulp flour in combination with tailored starter cultures can support the industrial production of functional breads with enhanced nutritional value, prolonged stability, and consumer-oriented sensory quality. Producers may consider a 10% carob pulp flour substitution to develop nutritionally enriched and commercially viable breads. Furthermore, promoting carob, a sustainable and underutilized crop, aligns with strategies for environmentally conscious, value-added bakery products. Therefore, further research is needed to identify the optimal microbial consortia for leavening carob-enriched bread, in order to enhance technological and nutritional performance, shelf life, and consumer acceptance.

## Figures and Tables

**Table 1:** Chemical composition (g/100 g) of carob pulp flour, wheat flour, and CB10 blend. Values are mean ± SD (n = 3). Different superscript letters within the same column indicate significant differences (*p* < 0.05).

Sample	Moisture	Protein	Ash	Fat	Fiber	Carbohydrates *
Carob flour	4.10 ± 0.01 ^c^	4.90 ± 0.30 ^c^	2.96 ± 0.02 ^a^	5.60 ± 0.04 ^a^	31.0 ± 0.2 ^a^	51.44 ^c^
Wheat flour	14.0 ± 0.01 ^a^	12.0 ± 0.02 ^a^	0.47 ± 0.04 ^c^	1.60 ± 0.05 ^c^	3.0 ± 0.3 ^c^	68.93 ^a^
CB10 blend	13.6 ± 0.02 ^b^	11.1 ± 0.03 ^b^	0.72 ± 0.01 ^b^	2.00 ± 0.08 ^b^	5.8 ± 0.4 ^b^	66.91 ^b^

* Carbohydrates calculated by difference.

**Table 2:** Alveograph and Farinograph parameters of wheat flour and the CB10 blend.

	Farinograph Parameters			Alveograph Parameters			
Flours	WA	DDT	DS	P	L	P/L	W
	%	min	min	(mm H_2_O)	(mm)	(−)	(10^−4^ J)
**Wheat Flour**	56.3 ± 0.10	2.3 ± 0.09	11.3 ± 0.15	72 ± 1.20	129 ± 0.70	0.56 ± 0.15	279 ± 6.20
**CB10 blend**	56.5 ± 0.12	5.9 ± 0.10	7.7 ± 0.20	76 ± 1.40	125 ± 0.85	0.61 ± 0.10	236 ± 5.10

**Table 3:** Evolution of pH values in LB, LI, and LS doughs during fermentation (T0, T1, and T2) and the corresponding baked breads at t1 (after 16 h of storage).

Time Points	LB	LI	LS
**T0**	5.34 ± 0.02 A,a	5.11 ± 0.02 B,a	5.12 ± 0.02 B,a
**T1**	5.21 ± 0.02 A,b	4.65 ± 0.05 B,b	4.55 ± 0.05 B,b
**T2**	5.06 ± 0.04 A,c	4.05 ± 0.03 B,c	3.75 ± 0.04 C,c
**Bread (t1)**	5.12 ± 0.01 A,d	4.12 ± 0.05 B,c	3.68 ± 0.07 C,c

Different lowercase letters: significant changes over time within the dough batch (*p* < 0.05). Different uppercase letters: significant differences between dough batches at the same time point (*p* < 0.05).

**Table 4:** Colorimetric indices (mean ± SD) and total color variation (ΔE) of crust and crumb in LB, LS, and LI breads. Different lowercase letters in the same column indicate significant differences between breads (*p* < 0.05).

		L*	a*	b*	C*	h	ΔE
**Crust**	**LB**	36.90 ± 1.84 ^c^	+13.93 ± 0.49 ^a^	+18.17 ± 1.57 ^c^	22.90 ± 1.45 ^b^	52.48 ± 1.95 ^c^	
	**LI**	48.26 ± 1.77 ^b^	+10.52 ± 0.38 ^b^	+23.71 ± 0.96 ^a^	25.94 ± 1.02 ^a^	66.18 ± 0.34 ^b^	13.1
	**LS**	55.88 ± 2.18 ^a^	+8.70 ± 0.67 ^c^	+22.33 ± 0.51 ^b^	23.96 ± 0.65 ^b^	68.80 ± 1.31 ^a^	20.1
**Crumb**	**LB**	49.37 ± 2.09 ^a^	+8.66 ± 0.26 ^a^	+21.59 ± 0.60 ^a^	23.25 ± 0.62 ^a^	68.22 ± 0.51 ^a^	
	**LI**	44.10 ± 1.15 ^b^	+7.74 ± 0.19 ^b^	+19.65 ± 0.27 ^b^	21.11 ± 0.31 ^b^	68.58 ± 0.30 ^a^	5.7
	**LS**	44.00 ± 2.46 ^b^	+7.82 ± 0.36 ^b^	+19.35 ± 0.75 ^b^	20.87 ± 0.79 ^b^	68.08 ± 0.72 ^a^	5.6

**Table 5:** Changes in moisture content, water activity, loaf weight, loaf volume, and specific volume of LB, LI, and LS breads over 7 days of storage at room temperature.

Bread Batch	Parameter	t_1_	t_3_	t_7_	Δ (t_7_ − t_1_)	%Δ vs. t_1_
**LB**	Moisture (%)	30.7 ± 0.42 ^aB^	30.5 ± 0.60 ^aB^	22.6 ± 1.21 ^bB^	−8.1	−26.4%
	aw (−)	0.94 ± 0.00 ^aB^	0.92 ± 0.00 ^aB^	0.88 ± 0.00 ^bB^	−0.06	−6.4%
	Volume (mL)	667 ± 22 ^aB^	620 ± 8 ^bB^	507 ± 15 ^cB^	−160	−24.0%
	Specific vol. (mL/g)	2.21 ± 0.09 ^aB^	2.23 ± 0.05 ^aB^	1.90 ± 0.07 ^bB^	−0.31	−14.0%
**LI**	Moisture (%)	29.6 ± 1.87 ^aA^	28.4 ± 0.39 ^aA^	21.2 ± 0.76 ^bA^	−8.4	−28.4%
	aw (−)	0.91 ± 0.01 ^aA^	0.89 ± 0.20 ^aA^	0.89 ± 0.00 ^aA^	−0.02	−2.2%
	Volume (mL)	615 ± 24 ^aA^	610 ± 7 ^aA^	553 ± 18 ^bA^	−62	−10.1%
	Specific vol. (mL/g)	2.08 ± 0.10 ^aA^	2.21 ± 0.04 ^aA^	2.10 ± 0.09 ^aA^	+0.02	+1.0%
**LS**	Moisture (%)	30.9 ± 0.63 ^aB^	29.4 ± 0.54 ^aB^	22.0 ± 2.33 ^bB^	−8.9	−28.8%
	aw (−)	0.91 ± 0.00 ^aB^	0.90 ± 0.00 ^aB^	0.85 ± 0.02 ^bB^	−0.06	−6.6%
	Volume (mL)	615 ± 20 ^aA^	608 ± 20 ^aA^	554 ± 21 ^bA^	−61	−9.9%
	Specific vol. (mL/g)	2.08 ± 0.08 ^aA^	2.24 ± 0.05 ^aA^	2.06 ± 0.09 ^aA^	−0.02	−1.0%

Different lowercase letters: significant changes over time within the same bread batch (*p* < 0.05). Different uppercase letters: significant differences between bread batches at the same time point (*p* < 0.05).

**Table 6:** Texture analysis on LB, LI, and LS breads over 7 days of storage at room temperature.

Parameter		LB	LI	LS
**Hardness (g)**	t1	75.87 ± 11.44 A,a	172.63 ± 25.85 B,a	164.75 ± 14.48 C,a
	t3	97.62 ± 15.16 A,b	260.74 ± 34.50 B,b	239.16 ± 28.18 C,b
	t7	145.57 ± 27.79 A,c	424.42 ± 46.57 B,c	376.25 ± 64.78 C,c
**Springiness**	t1	0.808 ± 0.04 A,a	0.869 ± 0.02 B,a	0.886 ± 0.01 C,a
	t3	0.662 ± 0.10 A,b	0.812 ± 0.09 B,a	0.826 ± 0.03 C,b
	t7	0.649 ± 0.03 A,b	0.865 ± 0.17 B,a	0.780 ± 0.08 C,b
**Cohesiveness**	t1	0.726 ± 0.04 A,a	0.603 ± 0.06 B,a	0.694 ± 0.06 C,a
	t3	0.434 ± 0.05 A,b	0.437 ± 0.05 B,b	0.488 ± 0.06 C,b
	t7	0.454 ± 0.06 A,b	0.345 ± 0.05 B,c	0.440 ± 0.09 C,b
**Gumminess (g)**	t1	54.94 ± 7.76 A,a	103.57 ± 15.27 B,a	114.16 ± 12.03 C,a
	t3	42.64 ± 9.33 A,b	113.47 ± 14.63 B,b	116.62 ± 18.26 C,b
	t7	65.40 ± 10.82 A,c	147.22 ± 32.64 B,c	166.60 ± 48.07 C,c
**Chewiness (g)**	t1	44.52 ± 7.42 A,a	90.17 ± 14.54 B,a	101.09 ± 10.18 C,a
	t3	28.77 ± 9.33 A,b	92.41 ± 17.92 B,b	96.62 ± 17.27 C,b
	t7	42.53 ± 8.07 A,a	127.35 ± 39.41 B,c	128.52 ± 36.42 C,c

Uppercase letters (A, B, C) indicate significant differences between bread batches (LB, LI, LS) at the same time point (*p* < 0.05). Lowercase letters (a, b, c) indicate significant differences within each batch across different storage times (*p* < 0.05).

## Data Availability

The data presented in this study are available on request from the corresponding author due to privacy.
